# Prediction of hot spot areas of hemorrhagic fever with renal syndrome in Hunan Province based on an information quantity model and logistical regression model

**DOI:** 10.1371/journal.pntd.0008939

**Published:** 2020-12-21

**Authors:** Zixi Chen, Fuqiang Liu, Bin Li, Xiaoqing Peng, Lin Fan, Aijing Luo

**Affiliations:** 1 XiangYa School of Public Health, Central South University, Changsha, Hunan, China; 2 Key Laboratory of Medical Information Research(Central South University), Changsha, Hunan, China; 3 The Fifth People’s Hospital of Qinghai Province, Xining, Qinghai, China; 4 Hunan Provincial Center For Disease Control And Prevention, Changsha, Hunan, China; 5 Big Data Center of Geospatial and Natural Resources of Qinghai Province, Xining, Qinghai, China; 6 Geomatics Technology and Application Key Laboratory of Qinghai Province, Xining, Qinghai, China; 7 Natural Resources Remote Sensing Center of Qinghai Province, Xining, Qinghai, China; 8 The second Xiangya hospital of Central South University, Changsha, Hunan, China; University Colorado Denver, UNITED STATES

## Abstract

**Background:**

China’s “13^th^ 5-Year Plan” (2016–2020) for the prevention and control of sudden acute infectious diseases emphasizes that epidemic monitoring and epidemic focus surveys in key areas are crucial for strengthening national epidemic prevention and building control capacity. Establishing an epidemic hot spot areas and prediction model is an effective means of accurate epidemic monitoring and surveying. *Objective*: This study predicted hemorrhagic fever with renal syndrome (HFRS) epidemic hot spot areas, based on multi-source environmental variable factors. We calculated the contribution weight of each environmental factor to the morbidity risk, obtained the spatial probability distribution of HFRS risk areas within the study region, and detected and extracted epidemic hot spots, to guide accurate epidemic monitoring as well as prevention and control. *Methods*: We collected spatial HFRS data, as well as data on various types of natural and human social activity environments in Hunan Province from 2010 to 2014. Using the information quantity method and logistic regression modeling, we constructed a risk-area-prediction model reflecting the epidemic intensity and spatial distribution of HFRS. *Results*: The areas under the receiver operating characteristic curve of training samples and test samples were 0.840 and 0.816. From 2015 to 2019, HRFS case site verification showed that more than 82% of the cases occurred in high-risk areas.

**Discussion:**

This research method could accurately predict HFRS hot spot areas and provided an evaluation model for Hunan Province. Therefore, this method could accurately detect HFRS epidemic high-risk areas, and effectively guide epidemic monitoring and surveyance.

## Author summary

Hunan, the main epidemic area of HRFS in China. Hunan has had a cumulative incidence of 117,000 cases since 1963. During this time Hunan experienced two high incidence periods in the 1980s and 1990s. We used an Information quantity + Logistic regression model (I+LR model) to predict high-incidence and potential epidemic HFRS areas. Normalized difference vegetation index(NDVI)contributed most to HFRS risk. Per capita GDP, population size, land-use type, rainfall, elevation, and soil type were all factors found to influence HFRS risk. Our study is useful for risk prediction, prevention, and control of HFRS.

## Introduction

Hemorrhagic fever with renal syndrome (HFRS) is a natural focal disease that is transmitted by rodents [1], with Hantaan virus (HTNV) and Seoul virus (SEOV) as the two main types of infections in China [2,3]. It is grouped with Class B infectious diseases. There were more than 150,000 cases of infection in mainland China from 2005 to 2019, and the HRFS disease prevention and control situation is currently grim [4]. Hunan, as the main epidemic area of HRFS in China, has had a cumulative incidence of 117,000 cases since 1963, during which it experienced two high incidence periods in the 1980s and 1990s. After 2000, the incidence has stabilized at 500 cases/year[5], but it has shown an upward trend in recent years. Accurately ascertaining the spatial distribution pattern and epidemic intensity of HFRS, and carrying out surveyance and risk assessment in key areas and areas with unknown epidemic sources, are of great importance for implementing accurate disease prevention and control measures [6].

The source of infection of HFRS is mice, and the disease is transmitted to humans by aerosol of their urine and feces or food that they have contacted, reflecting close contact between humans and mice [7]. Meteorology, topography, vegetation, and other factors can significantly affect the spatial distribution and population density of HFRS host animals [8,9]. Social environmental factors, such as human socio-economic activities and changes in spatial aggregation of the population are also closely related to HFRS [10–12]. Traditional epidemiological methods, which use administrative divisions as research units to analyze the spatial pattern of cases, the changing trends in incidence rate, and the correlation with environments, cannot address modern requirements of disease prevention and control [13]. A niche model has been gradually applied for predicting the risk of infectious diseases in recent years [14,15]. The Genetic Algorithm for Rule Set Production niche model, based on H5N1 case data from Nigeria in 2006, successfully predicted the H5N1 transmission risk in West Asia [16]. A niche model based on a geographic information system predicted the risk of hantavirus infection in Shandong Province [17]. Previously, correlation of environmental impact factors with the transmission of HFRS in Changsha City was assessed, taking into account eco-geographical characteristics [18]. The transmission risk of HFRS in the Dongting Lake region from 2005 to 2010 was predicted by using the specific time niche model [19]. The niche model uses the spatial distribution of species and the data of environmental variables to establish the relationship of ecological needs of species, and maps the calculated results to different spatial and temporal environments to predict the spatial distribution of species. Thus, niche models based on environmental factors and spatial case data can predict the potential transmission risk of epidemic diseases and the predicted results have certain universality. In addition to the presence of virus-carrying rodents, the occurrence of HFRS cases is important because of the close contact between rodents and human beings. Therefore, it is not enough to consider only the habitat of rodents, but it is also important to consider the factors such as social and economic activities of human beings.

This study regarded HFRS case sites in Hunan Province in China as independent samples, extracted the environmental values corresponding to the spatial case sites from 2010 to 2014, and calculated the contribution of information to natural and socio-economic environmental values of case sites, based on the information quantity model. We then constructed a binary logistic regression model with case sites and non-case sites, explored the non-random relationship between HFRS and environmental variables, and eventually predicted the spatial distribution and epidemic intensity characteristics of HFRS in Hunan Province.

## Methods

### Introduction to the study area

Hunan Province is located in central-south China (24°38’-30°08’N, 108°47’-114°15’E), with a total area of 218,800 square kilometers, and 13 prefecture-level cities and one autonomous prefecture under its jurisdiction. The annual average temperature is 16–19°C, and the annual average precipitation is about 1,450 mm. The province’s resident population is nearly 70 million, and the annual gross output is about RMB 4,000 billion yuan. Hunan is located in the transition zone from the Yunnan-Guizhou Plateau to the Jianghan Plain. The topography is characterized by a horseshoe-shaped terrain with mountains on three sides, with the opening toward the north. There is a dense river network distributed throughout Hunan and the water system is developed. The province has a continental subtropical monsoon climate.

## Data collection

### Case data

**Fig 1:**
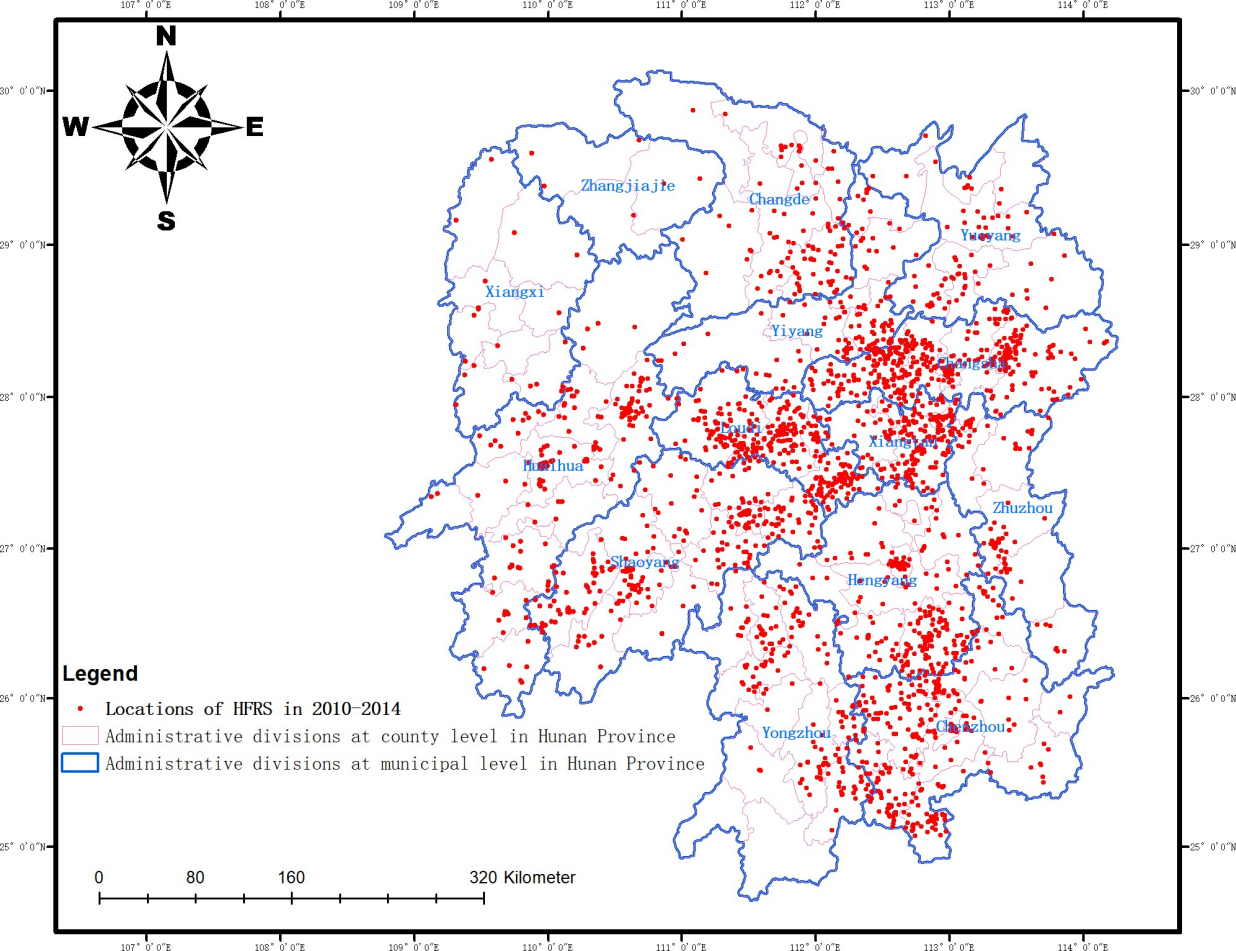
Distribution map of the HFRS case points in Hunan Province from 2010 to 2014.

Data on 3,128 HFRS cases from 2010 to 2014 and 3,388 HFRS cases from 2015 to 2019 were collected from the management system for reporting disease monitoring information of Hunan Provincial Center for Disease Control and Prevention (CDC, http://www.hncdc.com). This study did not distinguish between the HTNV and SEOV infection types. The Web Service API of Baidu Map was used to geocode the detailed current residence addresses in the case data (http://api.map.baidu.com/geocoding/v3/), and to analyze the spatial geographic coordinates and WGS84 coordinate system of the case sites, as shown in Fig 1.

### Non-case data

In order to satisfy the requirements of binary logistic regression analysis, 3128 non-case points were generated in the region without HFRS cases in Hunan province. The generation principle holds that the distance between the non-case points is greater than 1km, and the distance between the non-case points and the case points is greater than 2km.

### Data on administrative divisions

We included data on China’s provincial administrative divisions, and the city and county administrative divisions in Hunan Province.

### Environmental data

Environmental data included data on human socio-economic activities and natural geographic environments. The environmental data collected is shown in Table 1 below.

**Table 1:** Environmental data summary table.

Environmental factor	Socioeconomic activity data			Physical geographical environment data				
	Land Use	Population	Per capita GDP	NDVI	Temperature	Precipitation	Soil	Altitude

Considering that socio-economic environments vary little in the time domain but vary greatly in the space domain, this study collected land-use data, total gross domestic product (GDP) data, and total population data for 2010. Natural environments vary greatly in both the time and space domains. Therefore, the average normalized difference vegetation index (NDVI), average rainfall, and average temperature, from 2010 to 2014, were collected. There was a strong correlation between the total GDP and the total population; the per capita GDP data could be obtained by dividing the total GDP by the total population.

The land-use data in 2010 had a spatial resolution of 1 km and included six categories and 19 sub-categories. The data on population size and per capita GDP in 2010, as well as the data on averageNDVI, average temperature, and average rainfall from 2010 to 2014 had a spatial resolution of 1km. China’s soil classification data had a spatial resolution of 1 km, while the digital elevation model data of Hunan Province had a spatial resolution of 90 m.

**Fig 2:**
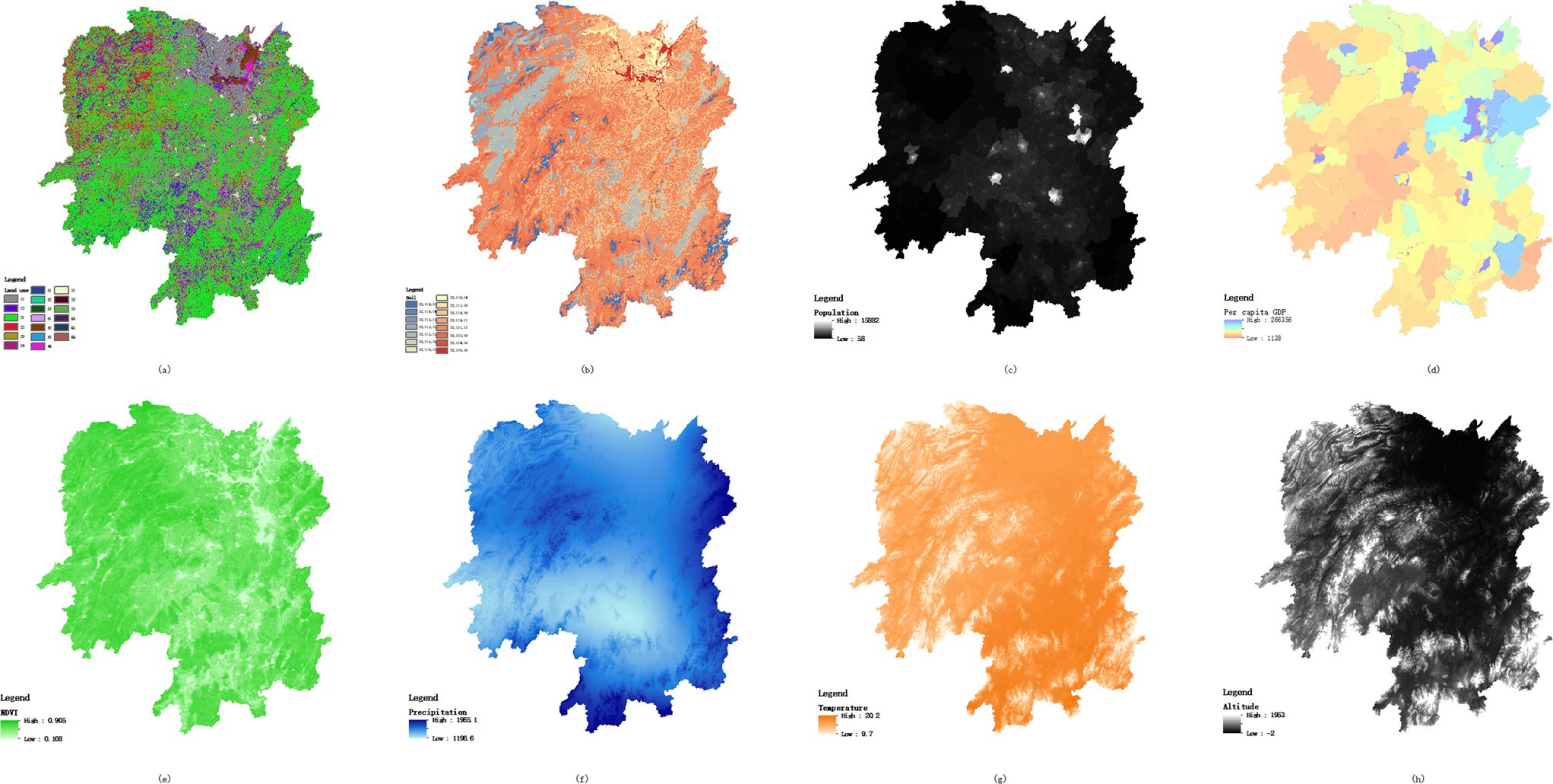
Spatial distribution of environmental impact factors in Hunan Province (a) Land use; (b) Soil; (c) Population; (d) Per capita gross domestic product (GDP) (e) normalized difference vegetation index (NDVI); (f) Precipitation; (g) Temperature; (h) Altitude.

The above data on administrative divisions and environments were all obtained from the Data Center for Resources and Environmental Sciences (http://www.resdc.cn/Default.aspx) of the Chinese Academy of Sciences, and the environmental data were subjected to projection transformation, resampling, etc. The coordinate system and spatial resolution were unified, and raster data were trimmed according to the boundaries of administrative divisions in Hunan Province, as shown in Fig 2 below.

### Analytical modeling

HFRS risk prediction was based on modeling research of the correlation between environmental factors and case data. Natural geographical environments affect the survival and population distribution of rodents, and change the viral infectivity to a certain degree, while human socio-economic activities are a prerequisite for the occurrence of HFRS; jointly, these determine the spatial aggregation characteristics and epidemic intensity distribution of HFRS [20,21]. HFRS mostly occurs in areas at relatively low altitudes, and the morbidity risk in cultivated land is relatively high [7]. Meteorological factors (including precipitation, temperature, etc.) have significant influences on seasonal changes in HFRS [22–24]. NDVI, land cover, elevation, and other environmental factors have important effects on the distribution of infectious rodents [25]. The selection of environmental factors for inclusion in models is affected by the accuracy of a priori knowledge, and collinearity and correlation issues may also exist among environmental variables. It is difficult to calculate the weight of environmental factors accurately when predicting epidemic disease risk areas with a single risk-assessment model. Risk prediction combining an information quantity model and a logistic regression model (*I+LR*) has been successfully applied in risk assessment of geological disasters [26]; however, this approach has not been applied to HFRS epidemic risk prediction previously, as we have done here.

By analyzing the activity radius of rodents and the precision of geocoded coordinates of case sites, and by considering the spatial resolution of prediction, Hunan Province was divided into 1km^2^ grid cells for risk area prediction. Summing up the natural geographical environment as well as human economic and social environment factors related to HFRS, this study used 2010–2014 as a research cycle and selected soil type, land-use type, temperature, rainfall, NDVI, terrain elevation, population size, and per capita GDP as environmental factor variables. The environmental values of case sites were extracted, and the classified information quantity contributed by the influence factors was calculated based on natural break classification and an information quantity model (*I*). The information quantity values were assigned to the case data and non-case data from 2010 to 2014, to build the sample sets, and 80% of the total sample sets were selected as training data to establish a binary logistic regression model. The collinearity and correlation characteristics of the influencing factors were analyzed, the weight coefficients of the environmental factors were recalculated after excluding significant influencing factors, and the 1-km-grid HFRS risk-prediction model for Hunan Province was constructed by superimposing environmental raster data using map algebra. ROC precision/recall curves were used to verify the prediction precision of the model for the training sample set and the test sample set. At the same time, case data from 2015 to 2019 were selected to check the rationality and accuracy of the predicted risk-zoning results, and to detect high-incidence and potential HFRS epidemic areas in the study area, in order to guide HFRS surveyance and risk assessment effectively.

### Selection and classification of environmental factors

In-depth understanding of the contribution by each environmental factor to the occurrence of cases and the cumulative effect among the factors is very important for the prediction and evaluation of HFRS risk areas as well as the improvement of zoning accuracy. According to the literature and expert experience [27,28], eight environmental factors were selected for inclusion in models, namely land-use category, soil category, population size in 2010, per capita GDP in 2010, annual average temperature in 2010–2014, annual average rainfall in 2010–2014, NDVI in 2010–2014, and elevation. Environmental values of the case sites were extracted and the distribution of each environmental factor value of the case sites was counted. Categorical environmental variables were classified by category, and continuous environmental variables were classified by range. Continuous environmental variables were classified by the Jenks natural breaks classification method, to minimize the variance of environmental values within a class and to maximize the variance of environmental values among classes. The land-use category was divided into 15 classes, the soil category was divided into 14 classes, and the remaining continuous environmental variables were classified into 15 classes. The classification of environmental factors is shown in Table 2.

**Table 2:** Table of classification and grading indexes of environmental impact factors.

Environmental factor	Classification number	Classification index
Land use	15	paddy field, dry land, woodland, shrubbery, sparse woodland, other woodland, grassland, river channel, lake, reservoir pond, beach, urban land, rural homestead, other construction land, unused land
Soil	14	yellow brown soil, red clay, lime (rock) soil, purple soil, stone soil, coarse bone soil, tidal soil, paddy soil, red soil, yellow soil, urban area, lakes and reservoirs, rivers, others
Population (people)	15	58~176,176~253,253~322,322~388,388~460,460~548,548~672,672~865,865~1132, 1132~1606,1606~2247,2247~3178,3178~4557,4557~7369,>7369
Per capita GDP (Ten thousand)	15	0.11~0.57,0.57~0.82,0.82~0.95,0.95~1.06,1.06~1.18,1.18~1.34,1.34~1.49,1.49~ 1.64,1.64~1.89,1.89~2.23,2.23~2.64,2.64~3.30,3.30~4.78,4.78~7.89,>7.89
NDVI	15	0.108~0.423,0.423~0.487,0.487~0.545,0.545~0.598,0.598~0.642, 0.642~0.677,0.677~0.70,0.702~0.723,0.723~0.741,0.741~0.756, 0.756~0.770,0.770~0.786,0.786~0.803,0.803~0.825,>0.825
Precipitation (mm)	15	1197~1249,1249~1287,1287~1317,1317~1344,1344~1371,1371~1398, 1398~1423,1423~1449,1449~1474,1474~1496, 1496~1525,1525~1562,1562~1609,1609~1678,>1678
Temperature (°C)	15	9.7~13.6,13.6~16.0,16.0~16.9,16.9~17.5,17.5~17.7,17.7~17.9, 17.9~18.1,18.1~18.3,18.3~18.518.5~18.7,18.7~18.9, 18.9~19.1,19.1~19.3,19.3~19.5,>19.5
Altitude (m)	15	-2~56,56~84,84~113,113~147,147~182,182~216,216~248,248~284,284~335, 335~402,402~481,481~571,571~739,739~1022,>1022

### Information quantity model

Environmental factor values are dimensionless variables with multiple types and ranges, and variable values should be dimensionalized before regression analysis. The information quantity model is based on information theory and is a statistical analysis method that is commonly used for assessment and prediction of geological disasters [29]. In this study, it was used for prediction and analysis of HFRS epidemic risk. Firstly, assuming that the natural and social environment conditions were consistent in the HFRS analysis cycle, the information contribution by classification factors was determined by counting the areas of classification environmental factors and the numbers of cases. The formula is as follows:

$$\text{I}=\sum_{\text{i}=1}^{\text{n}}I(X_{\text{i}}^{\text{j}},H)=\sum_{i=1}^{n}\text{ln}\left(N_{i}/\text{N}\right)/\left(S_{i}/S\right)$$ (1)

where $X_{\text{i}}^{\text{j}}$ is the classification environmental factor of the study area; I ($X_{\text{i}}^{\text{j}}$, H) is the information quantity contributed by environmental factor $X_{\text{i}}^{\text{j}}$ to HFRS occurrence; *N*_*i*_ is the number of HFRS cases occurring in the classification environmental factor $X_{\text{i}}^{\text{j}}$; *N* is the total number of HFRS cases in the study area; *S*_*i*_ is the area of classification environmental factor $X_{\text{i}}^{\text{j}}$; *S* is the total area of the study area; *I* is the comprehensive information quantity in the evaluated unit; and *n* is the total number of classifications of influencing environmental factors; j represents the different environmental impact factors, such as land use, soil and elevation; i is the number of grades corresponding to different environmental factors j. For this calculation, the known quantities of different environmental factors can be placed into Formula 1 for calculation, which will yield the hierarchy of values for the various environmental factors as shown in Table 3.

**Table 3:** Classification and Grading Information Quantity Contribution Value of Environmental Impact Factors.

Category		Land use	Soil	Population	Per capita GDP	NDVI	Precipitation	Temperature	Altitude
Grading information value	1	0.4836	-3.0311	-1.5332	-0.3207	2.3199	-0.1313	-2.1985	0.2314
	2	0.0648	0.0740	-0.7570	-0.6257	2.7225	-0.0001	-2.2285	0.8245
	3	-0.4829	-0.6070	-0.0426	-0.0543	1.9551	0.6304	-1.1720	0.6315
	4	-1.4243	-0.3903	0.2306	-0.3058	1.6932	0.0426	-0.7668	0.4922
	5	-0.2569	-1.3436	0.3674	0.0964	1.6262	0.1263	-0.2197	0.2582
	6	-0.2284	-0.5536	0.5643	-0.3985	0.7466	0.0580	0.3936	0.3842
	7	-1.4876	-0.2954	1.2759	-0.2649	0.8443	0.0324	0.1748	0.4997
	8	0.1933	0.5193	1.5171	0.3276	0.5035	-0.1504	0.2598	0.3608
	9	-0.8011	0.0683	2.1170	0.2177	0.4480	0.4931	0.7184	0.1505
	10	-0.4973	-2.2488	2.4106	-0.4569	0.3278	0.1742	0.8609	-0.5692
	11	-1.1959	2.3453	1.7297	-0.1538	0.2264	-0.1125	0.6582	-0.9644
	12	3.0225	-1.0936	1.9941	1.2551	-0.0285	-0.4837	-0.0485	-1.1996
	13	0.9272	-0.5156	1.6439	0.4517	-0.3026	-0.2612	0.3209	-1.8827
	14	1.4870	0.0000	2.4580	1.1289	-1.1745	-0.5952	0.6620	-2.6316
	15	-1.0801		2.7685	1.3000	-2.3704	-2.0338	1.1716	-3.0381

### Logical Regression Model

Binary logistic regression is a generalized linear regression algorithm with dichotomous dependent variables [30]. In this study, it was used for statistical analysis of the relationship between epidemic status and the influencing factors of multiple environmental variables [31]. In the prediction of epidemic occurrence, a logistic regression model was used to analyze the relationship between binary dependent variables (0 means no occurrence, and 1 means occurrence) and environmental factor independent variables $\left(X_{\text{1}}^{\text{j}},X_{\text{2}}^{\text{j}},\cdots ,X_{\text{n}}^{\text{j}}\right)$ In this study, a case site was defined as 1 and a non-case site was defined as 0. The logistic regression function is as follows:

$$\{\begin{array}{c} Z=\beta_{0}+\beta_{1}x_{i}^{landuse}+\beta_{2}x_{i}^{soil}+\cdots +\beta_{n}x_{i}^{altitude}\\ P\left(Y=1|X\right)=1/(1-\exp\left(-Z\right) \end{array}$$ (2)

where p is the occurrence probability of the HFRS epidemic, and the value range is [0,1]; and *β*_*i*_ is the logical regression coefficient, $x_{i}^{landuse}$ is the information quantity of environmental factors of land use classification corresponding to the sample point. In this study, 80% of the 6,248 sample sites (including 3,124 case sites and 3,124 non-case sites) from 2010 to 2014 were randomly selected as the training sample set and the remaining 20% as the test sample set. The training sample and its corresponding grading information for environmental factors were put into Eq 2 to calculate the regression coefficient of the various environmental impact factors. Using the map algebra method to overlay various weighted environmental raster data, the Z value was calculated for the 1-km grid in Hunan province, and the probability map of risk areas in Hunan province after normalization was created.

Before logistic regression modeling, it is necessary to analyze whether there is multicollinearity among environmental variables, as high collinearity among variables will cause distortion in model estimation. Collinearity assessment was performed in the training sample set, and the variance inflation factor (VIF) was calculated to judge whether there was collinearity among environmental variables. If the VIF value was greater than 10, it suggested serious collinearity among environmental factors. As shown in Table 4, there was no serious collinearity among environmental variables. Further correlation analysis of the variables showed that the correlation coefficient between elevation and temperature variables was -0.589. After temperature was excluded as a variable, the remaining factors were comprehensively analyzed for independence. The correlations among all factors were below 0.3, and the environmental variables met the requirements for independence. Table 5 shows the correlation matrix. Logistic regression was performed to determine the weight values of the environmental factors. The significance index (sig) was used to judge the rationality of environmental variable selection. A sig value greater than 0.05 indicated that the environmental factor had no statistical significance. The results of HFRS binary logistic regression analysis are shown in Table 6 below. The sig values of environmental factors were all less than 0.05, indicating that the factors played a positive role in the model. The Wald statistic was used to test the significance level of the partial regression coefficient. It is a function related to the partial regression coefficient and the degree of freedom and is subject to chi-square distribution of which degree of freedom is 1. The larger the test value, the smaller the p value and the more significant the coefficient. In this study, it was used to evaluate the significance of the environmental variable coefficient.

**Table 4:** Variance inflation factor (VIF) values of the environmental factors.

Evaluation factor	Land use	Soil	Population	Per capita GDP	NDVI	Precipitation	Temperature	Altitude
VIF	1.523	1.459	2.089	1.145	2.646	1.119	3.191	3.331

**Table 5:** Correlation coefficient matrix of the environmental factors.

	Constant	Soil	Land use	Population	Altitude	NDVI	Precipitation	Per capita GDP
Constant	1.000	-0.172	0.127	0.067	-0.066	-0.006	-0.019	0.105
Soil		1.000	-0.044	-0.099	-0.305	-0.002	-0.011	-0.008
Land use			1.000	-0.092	-0.045	-0.201	-0.015	0.048
Population				1.000	-0.189	-0.278	-0.100	-0.007
Altitude					1.000	-0.297	0.013	-0.201
NDVI						1.000	-0.062	0.040
precipitation							1.000	-0.034
Per capita GDP								1.000

**Table 6:** Results of binary logistic regression modelinganalysis.

Evaluation factor	B	S. E.	Wals	df	Sig	Exp(B)
Soil	0.233	0.080	8.596	1	0.003	1.263
Land use	0.435	0.060	52.525	1	0.000	1.545
Population	0.437	0.048	83.647	1	0.000	1.548
Altitude	0.221	0.056	15.872	1	0.000	1.248
NDVI	0.563	0.045	156.431	1	0.000	1.755
Precipitation	0.435	0.090	23.447	1	0.000	1.545
Per capita GDP	0.810	0.082	96.971	1	0.000	2.249
Constant	0.082	0.036	5.125	1	0.024	1.085

Note: B represents the regression coefficient of each factor in the model; S.E. is the standard error; Wals is the Wald test statistic; df is degree of freedom; Sig indicates significance; Exp(B) is the odds ratio.

## Results

### Accuracy test

**Fig 3:**
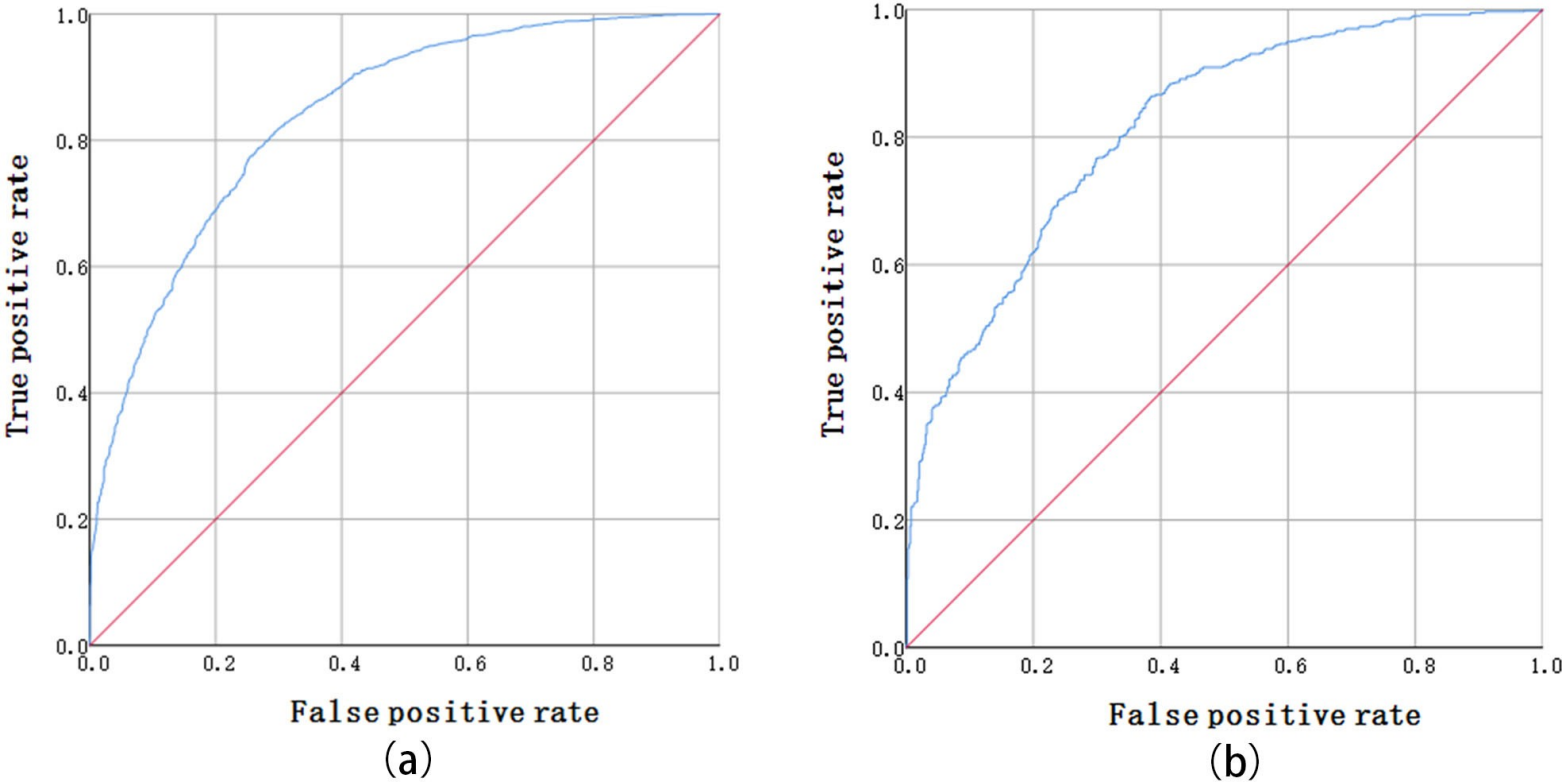
ROC curves of training samples (A) and test samples (B).

ROC curves are commonly used to verify the accuracy of predictions. In this paper, the ROC curve was selected to evaluate the accuracy of the HFRS risk-prediction model. The area under the ROC curve (AUC) was a measure of consistency or statistics similar to a binary model, used to evaluate the performance of a binary model. The closer the value is to 1, the closer the model prediction value and sample value [29,32]. The 2010–2014 training sample and test sample sets were tested for risk area modeling, as shown in Fig 3. The vertical axis in the curve represents the true-positive rate, i.e. the cumulative percentage of the actual incidence value; the horizontal axis represents the false-positive rate, i.e., the cumulative percentage of the predicted incidence probability value. The AUC value of the training data set was 0.840 (95% confidence interval [CI]: 0.829–0.850, SD = 0.005, P < 0.01), and that of the test data set was 0.816 (95% CI: 0.793–0.839, SD = 0.012, P < 0.01), indicating that the model had a good prediction effect. In this paper, an *I+LR* model was used to predict the HFRS risk areas in Hunan Province. The ROC precision test showed that the method could effectively screen the HFRS environmental factor variables and make accurate predictions about the environments in which HFRS occurs.

**Fig 4:**
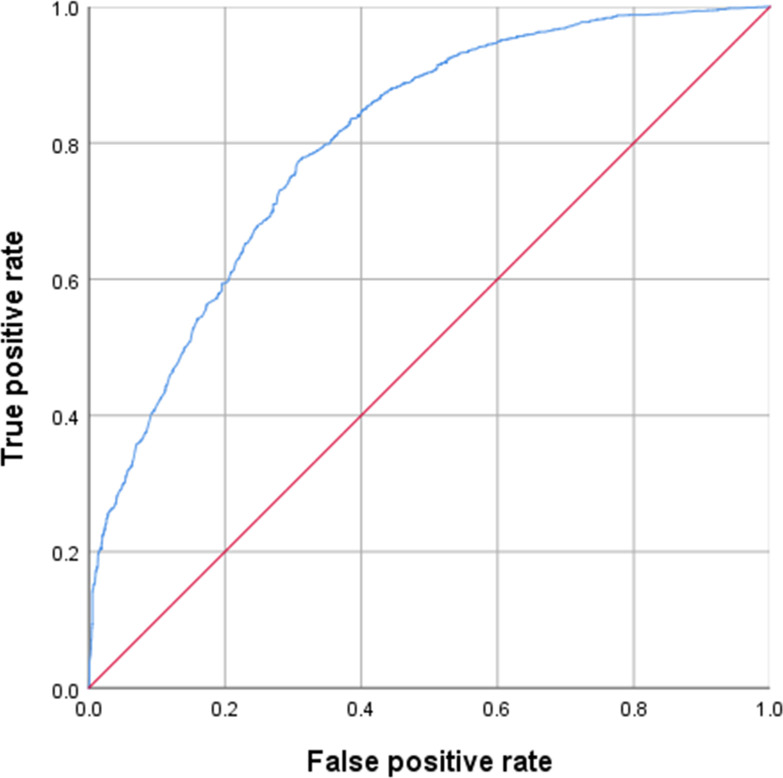
ROC curve of validation data set from 2010–2014.

As a novel epidemiological modeling and an analysis method, I+LR model was applied to the prediction and evaluation of HFRS hotspots. In order to conduct a more rigorous analysis of this method, this paper adopted the traditional binary logistic regression model based on “optimal binning” for precision comparison. First, a validation data set was established for the data of 6,248 cases from 2010 to 2014, and the environmental factor value of the case data set was preprocessed by the “optimal binning” method. The maximum binning threshold was set at 15. The continuous variables were divided into binning according to the threshold value directly. Category variables were divided into reasonable binning according to the number of categories, as shown in Table 7. To complete the “optimal binning” of environmental factors into the logistic regression model calculation analysis, land use, and average rainfall of SIG were assessed. The values were 0.504 and 0.192,respectively, (>0.05) indicating that the environmental factors value was not statistically significant. After excluding land use and the average rainfall value, a binary logistic regression calculation was performed again, and the AUC value of the data set was 0.800. The ROC curve is shown in Fig 4.

**Table 7:** 2010–2014 Environment factor binning value of data set.

Evaluation factor	Land Use	Soil	Population	Per capita GDP	NDVI	Precipitation	Temperature	Altitude
N	3	4	5	6	6	3	4	5

Compared with the traditional logistic regression model based on "optimal binning", the I+LR model improve the training accuracy by 4%. At the same time, the I+LR model can more reasonably solve the problem of nonlinear data discretization, better establish the relationship between environmental factors and the onset state, and better adapt to the modeling of various complex environmental variables.

### Risk zoning

**Fig 5:**
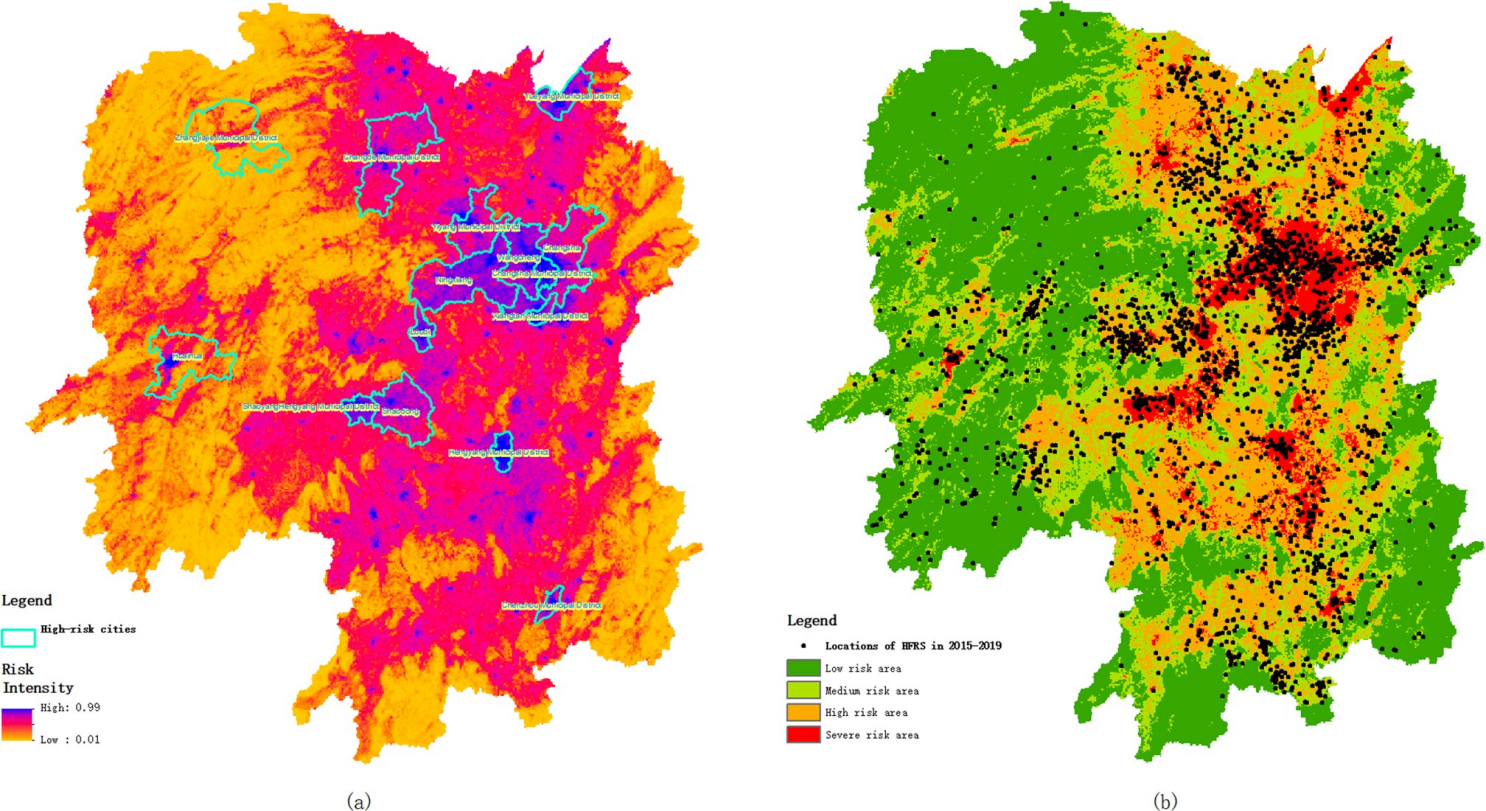
HFRS risk intensity map (a) and risk zoning map (b) of Hunan Province.

According to the weight coefficients of the influence factors obtained by the logistic regression model, the environmental raster data of the study area were superimposed to calculate the HFRS risk prediction probability for Hunan Province by map algebra. The risk prediction results were equally divided into four areas with severe, high, medium, and low risks according to the magnitude of probability values, generating the HFRS transmission risk zoning map of Hunan Province. The HFRS case data from 2015 to 2019 were superimposed, and the HFRS risk prediction zoning for Hunan Province was further evaluated. High risk areas are concentrated in the main urban areas of Changsha, Shaoyang, Loudi, Hengyang, Yiyang, Chenzhou, Xiangtan, Changde and Yueyang in the central and eastern part of Hunan Province. Ningxiang County and Shaodong County in the central and western regions along with Wangcheng County and Changsha County also have a high incidence. The incidence risk of HFRS was found to be low in the mountainous areas in the west and southeast of Hunan Province, and the modeling analysis results are consistent with the HFRS disease surveillance data of Hunan Provincial Center For Disease Control and Prevention. This modeling study found that the main urban areas of Huaihua city and Zhangjiajie city in western China were at greater risk of HFRS infection, which required high attention and active prevention and control measures. The range of HFRS disease hotspots in Hunan province is shown in Fig 5.

The relationship between the number of HFRS cases and the risk zoning level is shown in Table 8. The prediction results showed that the area ratio of severe and high risk areas were 8.25% and 26.15%, respectively, the rate of total cases was 42.68% and 39.43%, respectively. The density of cases in severe-risk areas reached 83.01 cases/100 km^2^, while that in high-risk areas reached 24.21 cases/100 km^2^. The risk partition diagram (b) is shown in Fig 5. This HFRS risk prediction zoning could accurately explain the case distribution and case density from 2015 to 2019, providing accurate geographic information support of epidemic intensity and spatial distribution for precise HFRS disease control, and assisting in the optimal allocation and accurate delivery of disease control resources.

**Table 8:** Relationship Between HFRS Case Quantity and Hot Spot Areas Level in 2015–2019.

Risk division level	Number of HFRS in 2015–2019	Area (1000km^2^)	Proportion of Total cases((%)	Risk area ratio(%)	HFRS Density (cases/100km^2^)
Severe risk area	1446	17.420	42.68	8.25	83.01
High risk area	1336	55.190	39.43	26.15	24.21
Medium risk area	440	47.579	12.99	22.54	9.25
Low risk area	166	90.882	4.90	43.06	1.83

Based on the HFRS case data and environmental factor data from 2010 to 2014, this study used the combination of an information quantity model and a logistic regression model to model and predict the risk probability of HFRS in Hunan Province, and used the HFRS case data from 2015 to 2019 to complete the rationality evaluation of HFRS risk zoning for Hunan Province. Among the environmental factors, NDVI contributed the most to HFRS morbidity risk, followed by per capita GDP, population size, land-use type, rainfall, elevation, and soil type. The accuracy of the logistic regression model was verified by ROC curve analysis. The AUC value of the training data was 0.840 and that of the test data was 0.816, indicating that the model had a high prediction level. The HFRS case data from 2015 to 2019 were superimposed on the risk prediction zoning map. The case sites in high-risk and severe-risk areas reached 82.1%, and all HFRS hotspot transmission areas in Hunan were detected, indicating that the HFRS risk prediction model had a high prediction ability for epidemic intensity and spatial distribution, and could explain the HFRS case distribution in Hunan Province well.

## Discussion

HFRS is a typical natural focal disease. Its epidemic distribution is significantly affected by natural geographical environments as well as by human social and economic activities, which reflects the close contact between human and rodents carrying pathogens. By analyzing and screening the environmental factors influencing the HFRS transmission risk, we applied an *I+LR* model to establish the non-random relationship between case data and environmental variables in the study area. We used this model to predict the transmission intensity and spatial pattern distribution of potential HFRS risk, to detect high-incidence and potential epidemic areas in advance, and to guide standardized and effective epidemic prevention management. This has important implications for disease prevention and control as well as for public health, and can provide a basis for research on risk prediction of similar epidemic diseases.

Among environmental factors, NDVI reflects the surface vegetation coverage, and is a comprehensive indicator for natural environment as well as human social and economic activities. In this study, it made the largest contribution to HFRS risk. The higher the NDVI value, the higher the vegetation coverage rate, and the lower the intensity of human activities. The NDVI value and HFRS incidence were negatively correlated. Per capita GDP reflects the level of human social and economic activities. Our results on per capita GDP represent that HFRS transmission is a close contact process between human and rodent infection sources, and indicate that urbanization has a significant impact on HFRS occurrence [33,34]. Population density reflects the degree of human spatial aggregation and was positively correlated with the occurrence of HFRS. The higher the population density, the greater the risk of contracting HFRS. Moreover, moderate precipitation can create conditions for the spread of HFRS. HFRS mostly occurs in the precipitation range of 1,300–1,500mm. Cultivated land as well as urban and rural construction land are still high-risk areas for HFRS. Soil type and elevation affect rodent distribution and contribute to HFRS. Generally, natural geographical environment factors affect rodent density and population distribution [35,36]. Active human social and economic activities are decisive conditions for the occurrence of HFRS. Human urbanization as well as close social and economic activities increase the risk of contact between people and infection sources, thus increasing the risk of HFRS transmission.

A classified selection of environmental factors is the focus of this study. Categorical variables were classified according to the number of HFRS cases for different types of variables, and continuous variables were classified according to the clustering of different environmental values of HFRS. In this study, dividing environmental variables into 15 classes for logistic regression modeling could provide good precision.

In binary logistic regression modeling, the 0-state indicated randomly extracted non-case sites, and the selection of non-case sites had a significant influence on the prediction accuracy and risk analysis of the model. If the threshold distance from the case sites was too short, a relatively low model prediction accuracy and a relatively small risk prediction zoning would be obtained. If the threshold distance was too long, a relatively high model verification accuracy and a relatively wide risk prediction zoning will be obtained. In this study, 2 km was selected as the threshold distance to generate non-case sites for establishing a regression model, which provided high model accuracy and reasonable risk prediction zoning results.

This study had some limitations. In this paper, biological factors were not considered when selecting environmental variables; however, biological factors, such as the density of mice as the main source of infection and the rate of virus presence in mice [33], have a very important influence on the spread of infectious diseases. During the modeling and exploration of the intrinsic relationship between spatial distribution characteristics of HFRS and environmental factors, only the spatial scale was considered, while the relationship between HFRS infection risk areas and the spatiotemporal scale changes in environmental factors was not established [37]. These topics should be explored in future studies.

This study presents a new attempt to analyze and predict HFRS disease hotspot areas by using I+LR model. The nonlinear relationship between spatial case data and multi-source environmental data was constructed through the combination model, which provided information on the incidence of HFRS with a 1km grid resolution in Hunan province. The results of the ROC curve showed that this method has good prediction accuracy, and the prediction zoning results could reflect the HFRS risk spatial distribution accurately for Hunan Province. A comparative analysis and accuracy verification of the traditional logistic regression model of "optimal binning" has also been performed in this study. In the next stage, the predictive performance of I+LR model will be further discussed. Other models (such as the BRT model) will be selected for comparative analysis [38], and the advantages and disadvantages of each model will be compared and discussed in detail.

In conclusion, we applied an *I+LR* model to establish the non-random relationship between case data and environmental variables in the study area to predict the transmission intensity and spatial pattern distribution of potential HFRS risk, in order to detect high-incidence and potential epidemic areas in advance. This approach has not been described for HFRS previously, and our findings have important application in disease prevention and control and can form a basis for research on risk prediction of other epidemic diseases.
